# Interventions to prevent unintended pregnancies among adolescents: a rapid overview of systematic reviews

**DOI:** 10.1186/s13643-023-02361-8

**Published:** 2023-10-19

**Authors:** Sahra Mohamed, Michael G. Chipeta, Tony Kamninga, Lomuthando Nthakomwa, Chimwemwe Chifungo, Themba Mzembe, Ruth Vellemu, Victor Chikwapulo, Maame Peterson, Leyla Abdullahi, Kelvin Musau, Kerri Wazny, Eliya Zulu, Nyovani Madise

**Affiliations:** 1 African Institute for Development Policy (AFIDEP), 13/41 Presidential Way, Public Service Pension Fund Building, P.O Box 31024, Lilongwe, Malawi; 2 Equity and Social Policy, ODI, London, UK; 3 The Children’s Investment Fund Foundation, Nairobi, Kenya; 4 The Children’s Investment Fund Foundation, London, UK

## Abstract

Risks associated with unintended pregnancy include unsafe abortions, poor maternal health-seeking behaviour, poor mental health, and potentially, maternal and infant deaths. Adolescent girls with unintended pregnancies are particularly vulnerable as they are at higher risk of eclampsia, premature onset of labour, and increased neonatal morbidity and mortality. Unintended pregnancy, with the right combination of interventions, can be avoided. Evidence-based decision-making and the need for a robust appraisal of the evidence have resulted in many systematic reviews. This review of systematic reviews focuses on adolescent pregnancy prevention and will seek to facilitate evidence-based decision-making. Two review authors independently extracted data and assessed the methodological quality of each review according to the AMSTAR 2 criteria. We identified three systematic reviews from low- and middle-income countries and high-income counties and included all socioeconomic groups. We used vote counting and individual narrative review summaries to present the results. Overall, skill-building, peer-led and abstinence programmes were generally effective. Interventions focused on information only, counselling and interactive sessions provided mixed results.

In contrast, exposure to parenting and delaying sexual debut interventions were generally ineffective. Adolescent pregnancy prevention interventions that deploy school-based primary prevention strategies, i.e. strategies that prevent unintended pregnancies in the first place, may effectively reduce teenage pregnancy rates, improve contraceptive use, attitudes and knowledge, and delay sexual debut. However, the included studies have methodological issues, and our ability to generalise the result is limited.

## Background

Sub-Saharan Africa’s (sSA) adolescent population (aged 10–19) is growing [8]. Child mortality on the continent is declining faster than fertility, which has increased the relative proportion of the adolescent population [44]. Consequently, many countries enter a new demographic era that allows them to leverage this youthful population to ensure a favourable population structure for social and economic gains [13]. For countries to reap these social and economic benefits, targeted investments in adolescents’ health, education, and well-being must be sustained. However, as of 2019, adolescents aged 15–19 years in low- and middle-income countries (LMICs) experienced an estimated 21 million unintended pregnancies per year, resulting in an estimated 12 million births [21], Sully et al. [67]. Over half (55%) of the unintended pregnancies among 15–19-year-old adolescent girls result in abortions, which are frequently unsafe in LMICs [77].

The potential consequences of unintended adolescent pregnancies are well known. They include adverse developmental, economic and health outcomes such as child undernutrition, increased risk of school dropout rates and decreased educational attainment [24, 74]. Adolescents are more likely to be discriminated against when seeking information and services related to sexual and reproductive health because they may feel embarrassed or encounter judgmental providers and be stigmatised [10, 62]. In contexts where contraceptive and abortion services are unavailable, difficult to access or illegal, this can result in women opting for and receiving sub-standard or unsafe services. These risks are heightened for adolescent girls and can lead to long-term adverse health impacts and death [20].

Early childbearing is also associated with risks for adolescent girls who continue the pregnancy. Compared to older mothers, adolescent mothers are far more likely to deliver prematurely, suffer complications during labour, and give birth to a low-birth-weight baby [6]. Moreover, children born to adolescent mothers are far more likely to be stunted, wasted or underweight [74].

Pregnant adolescents and adolescent mothers drop out of school at much higher rates than their non-pregnant peers, even in countries with policies encouraging pregnant adolescents to remain in education [66]. A lack of education limits girls’ economic and social opportunities, increasing their dependency on others and, in turn, their vulnerability. Adolescent pregnancy is a public health problem. Sustainable Development Goal (SDG) 3.7.2, which focuses on the adolescent birth rate, acknowledges this and tasks the global community to address this issue. In sSA, approximately one in five teenage girls become pregnant [35]. While the overall trends in adolescent birth rates show a decline globally, the sSA region continues to exhibit a significant lag [71].

The COVID-19 pandemic has exacerbated this public health problem by interrupting access to essential health services [35]. In addition to health services being interrupted, other essential services, such as schools, were also affected. There is an evidence base for the protective effect schooling has on adolescent girls, as it decreases their likelihood of becoming a child bride and falling pregnant [1, 40, 56]. Access to adolescent-friendly sexual and reproductive health services is critical for girls who find themselves pregnant. Despite this, many girls have been unable to attend school and access sexual and reproductive health services due to national lockdowns and severe restrictions on movement, significantly increasing their chances of getting pregnant [40]. As national governments seek not only to address the virus but also the consequences of the virus, such as an increase in child marriages, school dropouts and pregnancy, policymakers want to understand better what interventions work and how applicable these interventions are to their context [46, 79].

### Description of the condition

Unintended pregnancy is defined as a pregnancy that is either unwanted, such as a pregnancy that occurs when no children are desired, or a pregnancy that is mistimed, such as pregnancies that occur earlier than desired [61]. Approximately 40% of all global pregnancies are unintended, and a quarter occur in sub-Saharan Africa [7]. The issue is particularly acute among adolescent girls, with at least 12 million 15–19-year-old girls in the developing world dealing with unintended pregnancy [21]. Unintended pregnancies tend to occur when adolescent girls or their partners do not use family planning methods, use them incorrectly or inconsistently or are coerced into sex. Unintended pregnancy is not a static concept since pregnancy can start by being intended and then become unintended because of circumstances in which the adolescent finds herself. Many factors influence the different pathways that lead to an unintended pregnancy. This includes but is not limited to a lack of sexual and reproductive health (SRH) knowledge, access to adolescent-friendly SRH services and lack of resources to access services [78]. The inability to openly discuss and make contraceptive decisions has also been cited as a barrier for adolescent girls, particularly girls with older partners [35]. In certain situations, adolescent girls may be subjected to societal pressure to marry and have children once married. These situations or circumstances encompass social and peer pressures to engage in sexual activities, conceive, coercion exerted by familial influences, and limited autonomy concerning contraceptive selection and utilisation, contributing to adolescent pregnancy incidence [19]. However, it should also be noted that some adolescents intend and desire pregnancy and childbirth [25].

Unintended pregnancy is associated with an increased risk of unsafe abortions, poor maternal health-seeking behaviour, poor mental health and potential maternal and infant deaths [9, 34, 73]. A recent study compared children’s morality rates among first-time mothers aged <16 years, 16–17 years and 18–19 years and found they were about 2–4 times, 1.5–2 times and 1.2–1.5 times higher, respectively, than among children of mothers aged 23–25 [51].

Adolescent girls faced with unintended pregnancies are particularly vulnerable as they are at higher risk of eclampsia, premature onset of labour, and increased neonatal morbidity and mortality. Complications like pregnancy-induced hypertension (11.4 vs 2.2%, *p*<0.01), pre-eclamptic toxaemia (4.3 vs 0.6%, *p*<0.01), eclampsia (4.9 vs 0.6%, *p*<0.01) and premature onset of labour (26.1% vs 14.6%, *p*<0.01) occurred more commonly in teenagers compared to women aged 20–30 years old [37]. Unintended pregnancy, with the right combination of interventions, can be avoided. The right interventions must first address the economic, socio-cultural and environmental factors that place adolescents in a position to deal with an unintended pregnancy. It is also necessary to implement these interventions within a context that ensures girls can access essential health services and also addresses individual-level factors like education and self-esteem for girls to take control of their sexuality and utilise the services available to them. Thus, lowering teenage pregnancies overall has an impact on reducing unintended pregnancies among adolescents.

### Description of the intervention

For this study, we define intervention(s) as any activities that target adolescents and are undertaken to prevent unintended pregnancies among adolescent girls. Pregnancies can be prevented by encouraging adolescents to delay their sexual debut, countering child marriage practices, increasing uptake and continued use of contraception, and educating girls and boys on the risks of unintended pregnancies [52]. Drawing from the literature on evidence-based guidelines for preventing adolescent pregnancies [68], we identify three pregnancy prevention strategies.

- *Primary prevention strategies*: Include strategies that prevent unintended pregnancies in the first place. Examples are a supportive family environment, comprehensive sexuality education, contraception, and prevention and detection of sexual and gender-based violence.
- *Secondary management strategies*: Early pregnancy diagnosis and counselling on pregnancy options, including access to safe abortion care.
- *Tertiary management strategies*: Prevention of adverse events associated with unintended pregnancy, for example, treatment of incomplete abortion; access to services for psychosocial trauma; and services for antenatal care and maternity services to prevent maternal morbidity and mortality.

### How the intervention might work

Communities that tend to be the most successful adopt a multifaceted approach, i.e. implement prevention programmes that operate at the primary, secondary and tertiary levels. These programmes seek to not only focus on sex and the potential consequence of engaging in unsafe sex but also address contextual factors such as social norms, empowerment, skill training and personal development. Moreover, the target group should not be limited to adolescent girls but also include adolescent boys, parents, teachers and community leaders [52]. For this review, we focus on primary prevention strategies. For example, school-based programmes delivered via the school platform mainly focus on psychosocial risk and protective factors that involve sexuality. These primary prevention strategies aim to improve adolescent girls’ and boys’ knowledge and awareness of their sexual reproductive health and reduce unintended pregnancies [69].

Leveraging the school platform to deliver interventions helps ensure that students have a safe space to learn about their sexuality, pregnancy prevention and the transmission of sexually transmitted diseases, and where to access SRH services. Moreover, school-based programmes allow adolescents to engage with these topics in a socially acceptable forum. Delivering sex education via schools ensures that more adolescents are reached before their sexual debut [41].

Although clinic and community-based (i.e., establishing health clubs to educate on SRH and facilitate referrals to clinics, mobile health clinics for youth and SRH services within youth centres) tend to incorporate elements of educational programmes, unlike school-based interventions, these sessions can also be delivered separately as a stand-alone intervention both within the clinic or outside on the broader community, to include out of school adolescents. These interventions also promote access to family planning services for adolescents, improve adolescents’ knowledge of methods and dispel misconceptions [59]. Community contraceptive-promoting activities can also seek to shift social norms within the community that inhibit the uptake of methods, which also facilitates the acceptability of sex educational programmes at school, the creation of adolescent-friendly services and the promotion and distribution of methods [59].

Youth development interventions not only focus on the sexual health needs of the target population but also address these needs within a programme that tries to tackle other cross-cutting issues through skill building and mentorship. The success of these programmes is contingent upon the involvement of various stakeholders such as schools, religious groups, the community, health officials and adolescents. In addition, implementors must ensure the intervention is practical, culturally apt and evidence-based [52].

### Why it is essential to do this review

Evidence-based decision-making and the need for a robust appraisal of the evidence have resulted in many systematic reviews. Systematic reviews are a valuable tool for summarising a large body of evidence, and this is reflected in the statistics that approximately 22 new systematic reviews are published daily [29]. However, a large number of systematic reviews leads policymakers and other decision-makers to find themselves unable to call upon a single document that robustly apprises the current state of the evidence. Currently, many systematic reviews focus on adolescent pregnancy prevention. A decision-maker who wants to understand better what interventions they can implement to curb adolescent pregnancy rates would have to review multiple systematic reviews. A systematic review like this one aims to focus more broadly on an outcome, such as unintended adolescent pregnancy, and identify potentially effective interventions [4]. Thus, this review of systematic reviews focuses on unintentional adolescent pregnancy prevention and will seek to facilitate evidence-based decision-making.

### Objectives

To synthesise systematic reviews on interventions to prevent unintended adolescent pregnancy.

#### Specific objective

- To understand barriers to and enablers of interventions focused on adolescent pregnancy prevention.
- To identify the best practices and interventions to combat unintended adolescent pregnancy.

## Methods

The study’s synthesis method was adopted from the provisional recommendations from the Cochrane Rapid Reviews Methods Group [26]. This methodology is the Cochrane provisional recommendation for conducting a rapid review of systematic reviews. In addition, we also reviewed two papers that provided reporting guidelines for overviews of reviews [27, 54]. The study is registered with PROSPERO, registration number CRD42021266470.

### PICOST matrix

- *Population*: Adolescent girls 15–19 years of age.
- *Intervention*: Any primary prevention strategy that may lead to a reduction of unintended adolescent pregnancies. Unintended pregnancy is defined as a pregnancy that is either unwanted, such as a pregnancy that occurs when no children are desired, or a pregnancy that is mistimed, such as pregnancies that occur earlier than desired.
- *Study setting*: All global studies focus on adolescent pregnancy prevention.
- *Comparator*: No intervention targeting to reduce unintended adolescent pregnancy over and above the ones listed under interventions.
- *Outcomes*: The outcomes include
  - ◦ *Primary outcome*: Unintended adolescent pregnancy
  - ◦ *Secondary outcomes*:
    - ◾ Use of contraception.
    - ◾ Change in knowledge of contraceptive effectiveness or effective method use.
    - ◾ Change in attitude towards contraception use.
- *Study design*
- The study included completed qualitative and quantitative systematic reviews.
- *Time*: Systematic reviews conducted between January 2015 and October 2022.

### Search methods for identification of studies

We searched for published systematic reviews in PubMed, Cochrane Library, and Epistemonikos published in English from January 2015 to October 2022. Medical subject headings and keywords used include “unintended pregnancy” and “adolescents” or include “unintended pregnancy” and “teenage” or “unwanted pregnancy among adolescents” or “mistimed pregnancy among adolescents” or “unwanted pregnancy among teenagers” or “mistimed pregnancy among teenagers” or “unwanted childbearing among teenagers” or “mistimed childbearing among teenagers” or “unwanted childbearing among adolescents” or “mistimed childbearing among adolescents” from January 2015 to October 2022 and “systematic review.” The search strategy used in PubMed was replicated in the other databases and is presented in Appendix 1.

### Data collection and analysis

#### Selection of studies

We adopted a 2-step screening process. In the first step, two authors (SM and MGC) and (TK and CC) independently screened the abstract of the studies retrieved from the electronic databases. After that, a full text of eligible studies was obtained for further review and the final selection of eligible studies for analysis. All the articles that did not meet the inclusion criteria were eliminated, and the reviewer indicated the reasons for elimination. Any disagreement that surfaced during the review was solved by a third party (either LA or LN) after thorough discussions on the issue.

#### Data extraction and management

Using a modified COCHRANE collaboration data extraction form Cochrane Effective Practice and Organisation of Care [14], we extracted and entered data from all study articles that met the inclusion criteria. The form also guided reviewers on extracting and recording data for uniformity. The following details were extracted from the included systematic reviews:

1. Name of the first author
2. Publication year
3. Location of the study
4. Data collection period
5. Adolescent pregnancy prevention interventions
6. Analysis methods

### Quality assessments

#### Assessment of methodological quality of included reviews

We assessed the methodological quality of each systematic review using AMSTAR 2: “A Measurement Tool to Assess Reviews 2” [64]. The AMSTAR 2 is a critical appraisal tool with 16 criteria to evaluate the quality of systematic reviews of randomised controlled trials. For this review, we grouped the bottom (scores 0 to 4), middle lower (scores 5 to 8), middle-upper (scores 9 to 12), and upper (13–16) quartiles. Two review authors independently performed quality assessments (SM and MGC), and discussion between review authors resolved disagreements.

#### Quality of the evidence in included reviews

We extracted information on the risk of bias (RoB) methods and ratings used in the included systematic reviews. In addition, where provided in the reviews, we extracted GRADE (Grading of Recommendations, Assessment, Development and Evaluations) ratings for the outcomes of interest for the review to assess the certainty of the evidence.

### Data synthesis

We organised the results according to (i) the type of adolescent pregnancy prevention intervention(s) and (ii) the type of outcomes being assessed. We only included studies that reported our primary outcome of interest, unintended adolescent pregnancy. Having viewed the outcomes reported, we categorised them into five groups: unintended adolescent pregnancy rates, improved contraception use, improved knowledge, improved attitude towards contraception use and delayed sexual debut. We used vote counting and individual narrative review summaries to present the results. We reported all outcomes reported by the studies within the relevant category (not preferencing one outcome over a similar or overlapping one). We then reported results as the number of outcomes favouring the intervention out of the total number of outcomes, based on the direction of effect and not necessarily statistical significance as suggested by the Cochrane Handbook for Systematic Reviews of Intervention [32].

## Results

**Fig. 1:**
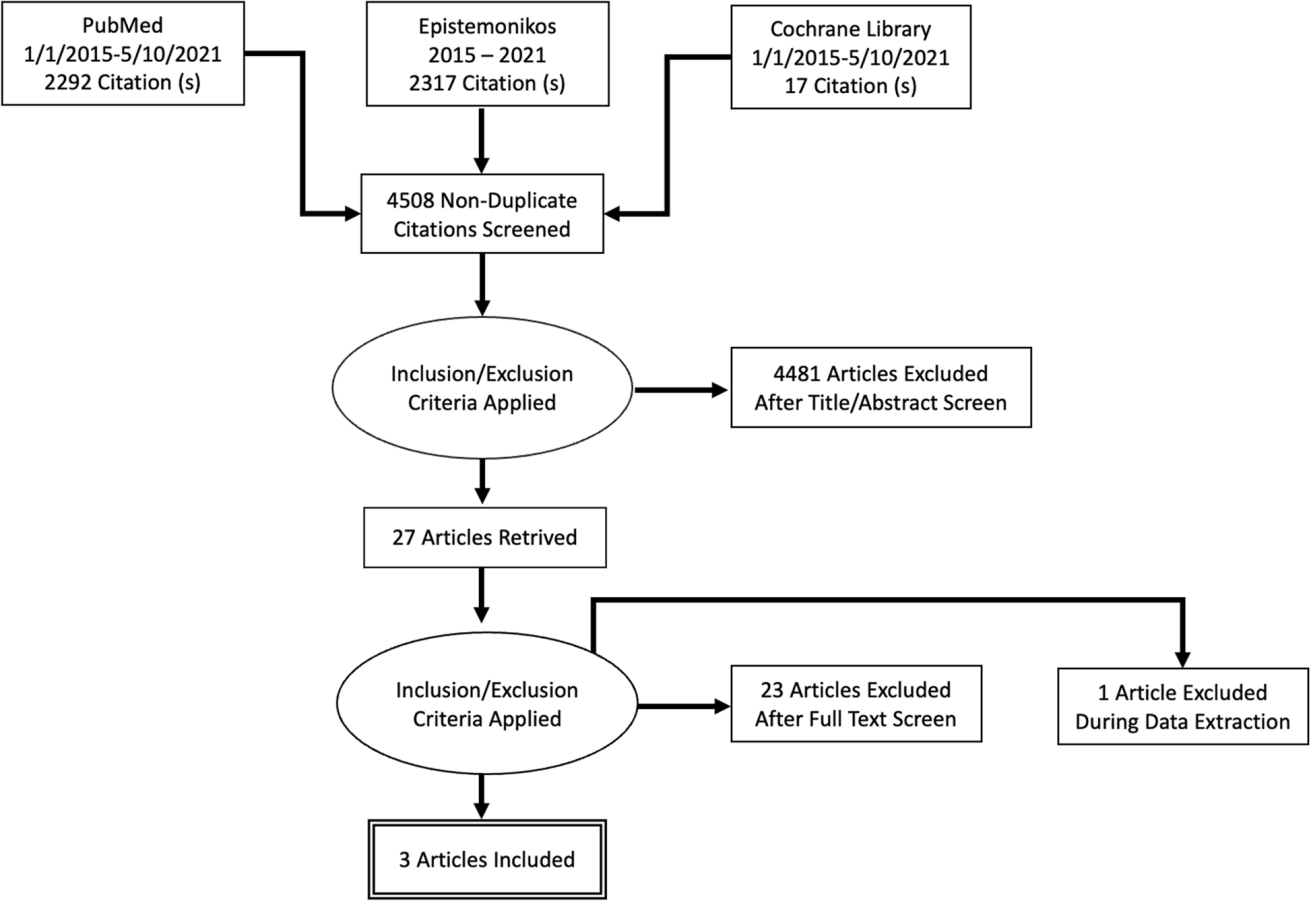
PRISMA flow chart for reviews identified and included in the review

Our initial electronic database search generated 4626 titles. After searching and removing duplicates, 4508 titles remained. During the first round of screening, we excluded 4481 titles and reviewed the remaining 27 titles in more detail. Of the 27, 24 were excluded for reasons elaborated in Appendix 2 and three studies were included in the overview of systematic reviews [39, 43, 52]. Figure 1 below elaborates on the study screening and selection process.

### Summaries of individual reviews

#### Lopez et al, [39]

Lopez et al. [39] stated that the aim was to ‘identify school-based interventions that improved adolescent contraceptive use’ [39] searched five databases. They also searched trial registries for recent trials. The study included twenty-one trials, but only the five studies that measured unintended pregnancy were included in this review. All five studies were cluster randomised control trials based in schools. The students were aged between 13 and 18 years. Four out of five studies occurred in the global north: two in the USA (Coyle [18], Kirby [36]) and two in the UK (Wight [76], Stephenson [65]) cited in [39, 69] South African study is the only exception. One study evaluated the effect of a school-based intervention that combined active learning, information provision, and skill development to reduce unsafe sexual behaviour and unwanted pregnancies and improve the quality of sexual relationships (Wight [76]), cited in [39]. Another study examined skills-based HIV, sexually transmitted Infections (STI) and pregnancy prevention curricula. It compared this to standard school-based activities related to the prevention of HIV, STI and pregnancy implemented by presenters from community-based agencies (Coyle [18]), cited in [39]. Another study addressed unwanted teen pregnancies holistically and looked at an interactive programme that addressed choice, body development, contraception and parenthood [69], cited in [39].

Two studies looked at peer-led interventions. The first peer-led interventions evaluated the impact of HIV (AIDS) and pregnancy prevention with activities focused on delaying intercourse and increasing contraception (Kirby [36]), cited in [39]. The second assessed a school-based peer-led sex education programme that focused on improving the quality of sexual relationships, STI and pregnancy prevention (Stephenson [65]), cited in [39].

#### Mason Jones et al. [43]

Mason Jones stated the aim was ‘to evaluate the effects of school-based sexual and reproductive health programmes on sexually transmitted infections (such as HIV, herpes simplex virus, and syphilis), and pregnancy among adolescents’ [43] searched six bibliographic and two conference databases. The study included twenty-one trials, but only the six studies that measured unintended pregnancy were included in this review. All six studies were cluster randomised control trials based in schools. The students were aged between 13 and 18 years of age. Three studies were in sub-Saharan Africa (Duflo [22], [59], Cowan [16]) cited in [43]. Two in Europe (Henderson [30], Stephenson [65]) cited in [43] and one in Latin America (Cabezón [12]) cited in [43].

Teachers delivered interventions in four studies. The first, Cabezón [12], cited in [43], evaluated the Teen STAR programme, stressing abstinence, fertility awareness, and the psychological and personal aspects of sexuality. Contraceptive use was not recommended. The second, Henderson [30], cited in [43], looked at the effect of a teacher-based programme that advised students to delay sexual intercourse and encouraged condom use. The third was Duflo’s [22], cited in [43] trial that evaluated a teacher-delivered programme promoting abstinence until marriage. Ross [59], cited in [43], reviewed a teacher and peer assistant-led programme to provide knowledge and skills to delay sexual debut, reduce sexual risk-taking and increase appropriate use of health services.

Peer educators delivered two studies. Cowan [15], cited in [43], was delivered by professional peer educators whose HIV prevention activities adapted the ‘MEMAkwa Vijana’ programme, which included modules focused on self-awareness, communication, self-belief and gender. This was delivered alongside programmes to improve communication between parents and children and increase support for adolescent reproductive health. Stephenson [65], cited in [43], a trial that looked at trained peer educators who delivered sessions that focused on sexual communication and condom use, knowledge about pregnancy, STIs (including HIV), contraception and local sexual health services.

#### Oringanje et al. [52]

The [52] review aimed to assess the effects of primary prevention interventions on unintended adolescent pregnancies. Oringanje et al. [52] searched ten electronic databases and three trial registers. The review contained fifty-three studies, but only eight that measured unintended pregnancy were included. Four were randomised control trials, and the remaining four were cluster randomised control trials. The study participants were aged between 12 and 19 years old. Five studies were in the USA (Herceg-Brown [31], Morrison-Beedy [48], Philliber [53], Howard [33], Kirby [36]) cited in [52]. For the remaining studies, Cabezon [12] took place in Chile, Wight [76] in Scotland, and Bonell [11] in England cited in [52].

All eight studies took a holistic approach to preventing unintended pregnancy. Four studies occurred within the school setting (Howard [33], Kirby [36], Stephenson [65], Cabezón [12]) cited in [52]. Cabezon [12], Howard [33] and Kirby [36] cited in [52] all delivered in-person sessions on health/STI education, skills building and contraceptive education. Similarly, Wight [76] cited in [52] delivered health/sex education, skills-building and contraceptive education. However, in this case, it was primarily delivered through interactive video.

### Summary across reviews

The included reviews reported results from 19 studies, of which four were included in more than one review (Cabezón [12]; Kirby [36]; Stephenson [65]; Wight [76]) cited in [39, 43, 52]. We did not remove the duplicates for this review but included them as individual trials. We had the following study designs: fifteen cluster randomised controlled trials and four individual randomised controlled trials.

### Population and settings

The primary target audience for all the studies included in the selected systematic reviews was adolescents. The age group started at 12–13 years; the overall upper limit was 19 years (Morrison-Beedy [48], cited in [52]). Three studies included male participants (Philliber [53], Kirby [36], Wight [76]) cited in [39, 52]. One study was unspecific (Howard [33] cited in [43, 52]). Sixteen out of 19 trials were in a school setting, and one was community-based. The setting was unclear for two studies included in this review (Morrison-Beedy [48], Philliber [53] cited in [52]). Eight trials were USA-based. Two trials were based in Chile, England, Scotland and the UK and one in Kenya, South Africa, Tanzania and Zimbabwe. Thirteen studies were conducted in high-, three in middle-, and three in low-income countries (LICs). Of the 19 studies included in the three reviews, four were conducted between 1986 and 1997, ten were between 2002 and 2008, and five were conducted between 2008 and 2015. All of the included reviews are at least 7 years old, and 74% were performed at least 10 years ago.

### Teenage pregnancy prevention interventions

We reviewed the nineteen studies and attempted to identify groups using the review author’s description. All interventions included sex education; therefore, studies were grouped based on additional intervention characteristics. We identified eight different adolescent pregnancy prevention intervention types or groups. All reviews did not contribute data to all categories but did contribute to at least one group.

#### Skills building

*Interventions that provide instruction, practice or other activities that are designed to help the target audience build and enhance their skills, i.e. teachers deliver better SRH classes or academic tuition for adolescents*.

Two reviews [39, 52] reported data from five different studies (Coyle [18], Howard, [33]; Kirby, [36]; Wight, [76]; Philliber, [53]).

Wight [76] (cited in [52]) identified teachers’ lack of sex education training as a barrier to the effective delivery of sex education classes. This paper investigated whether a teacher training intervention primarily delivered through an interactive video that combined active learning, information provision and skill development would improve adolescent SRH outcomes. Coyle [18] (cited in [39]) compared the effects of skills-based HIV, STD, and pregnancy prevention curriculum plus service-learning activities implemented 2 or 3 times per week for 5 to 7 weeks against usual activities related to the prevention of HIV, STI and pregnancy. Howard [33] (cited in [52]) also looked at a skill-building health/STD and contraceptive education intervention. Kirby [36] (cited in [52]) reviewed a classroom-based intervention that included health education, skills-building, contraceptive education and the standard sexuality curriculum. The team compared the impact of who delivered the sessions: teachers and young people. Philliber [53] (cited in [52]) looked at the impact of a wide range of skill-building activities, including but not limited to job clubs, academic skills, art and other recreational activities, as well as counselling, contraceptive education and access.

#### Interactive

*Interventions are based on a principle of student engagement, which requires a balance between student and teacher voices. Students and teachers are equally engaged in learning*.

One review [39] reported data from one study [69].

Taylor’s [69] (cited in [39]) intervention addressed concepts such as choice, body development and contraception using an interactive format.

#### Peer-led

*Interventions that use a method of teaching or facilitating health promotion that asks people to share specific health messages with members of their community*.

Three reviews [39, 43, 52] reported data from three studies (Cowan, [16]; Kirby [36]; Stephenson [65]).

Cowan [16] (cited in [43]) evaluated ‘professional peer educators’ (PPEs)—i.e. school leavers who were selected, trained, supervised and worked in the community for 8 to 10 months on SRH with adolescents. Kirby’s [36] (cited in [52]) was a peer-led HIV/AIDS and pregnancy prevention intervention with interactive activities that sought to delay intercourse and increase condom use. Stephenson [65] (cited in [39]) reviewed a school-based peer-led sex education project that included sexual communication, condom use, HIV/STI and different types of contraception, including emergency contraception and local sexual health services.

#### Delaying sexual debut

*Interventions seek to influence the timing or assist young people in delaying sexual initiation*.

One review [43] reported data from two studies (Henderson [30], [59]).

Henderson’s [30] SHARE (Sexual Health and Relationships: Safe, Happy and Responsible) programme (cited in [43]) trained class teachers on how to promote delayed sexual debut until they were ready and always use a condom until they planned to have children [59] (cited in [43]) examined teachers with peer assistants. The aim was to provide knowledge and skills to delay sexual debut, reduce sexual risk-taking and increase the appropriate use of health services.

#### Abstinence

*Interventions that actively discourage sex before marriage*.

Two reviews [43, 52] reported data from two studies (Duflo [22]; Cabezon, [12]).

Duflo [22] (cited in [43]) trained class teachers to deliver abstinence-focused sex education. Cabezon [12] [43, 52] examined an intervention that delivered a 45-min weekly class for a year on health education and skills-building but focused on abstinence and did not recommend contraceptive use.

#### Counselling

*Interventions that use talking therapy with a trained professional to help clients address their sexual and reproductive health needs*.

One review [52] reported data from one study (Herceg-Brown [31]).

Herceg-Brown ([31]) (cited in [52]) reviewed two interventions. The first was a family support group (regular clinic services plus 50 min of family or individualised counselling services on sex and contraceptive education for 6 weeks). This was compared to a periodic support group plus staff support through two to six telephone calls every 4 to 6 weeks after the initial clinic visit to monitor teenagers’ adjustment to contraceptives received at the clinic.

#### Exposure to parental responsibilities

*Interventions that expose adolescents to the realities of being a parent, i.e. childrearing*.

One review [52] reported data from one study (Bonell [11]).

Bonell [11] (cited in [52]) reviewed a study that delivered weekly 3-h sessions in preschool nurseries to develop an awareness of the responsibilities involved in parenting and build self-awareness and confidence to reduce the risk of teenage pregnancy.

#### Information only

*Interventions that focused on providing information only*.

One review [52] reported results from one study (individual RCTs).

### Duration, frequency and intensity of adolescent pregnancy prevention interventions

The duration of all included adolescent pregnancy prevention interventions was described for all 19 studies. The frequency, however, was only described for eight studies.

### Comparisons

The types of stated comparisons were (i) no intervention, (i) usual sex education/standard curriculum, (iii) compulsory life skill programme, (iv) youth programme and (v) general health promotion. In one study, the control intervention was not adequately described (Kirby [36], cited in [52]).

### Outcomes

The primary outcomes from the included reviews are summarised in Appendix 3.

#### Lopez et al. [43]

Two of the five included studies looked at adolescent pregnancy rates. Both interventions focused on skill-building. The remaining three studies examined self-reported “ever pregnant” or caused a pregnancy. Two of these studies focused on peer-led interventions. One of these peer-led interventions also examined abortion rates to deduce pregnancy rates. The third was an interactive programme that focused on choice and body development. Self-reported unwanted pregnancy was another outcome measured in two studies examining peer-led and skill-based interventions separately.

#### Mason Jones et al. [43]

All six papers included in this review looked at pregnancy prevalence or current pregnancy. Two papers investigated abstinence, two looked at peer-led interventions, and two focused on delaying sexual debut. Another outcome this review explored was “has been pregnant.” Two papers looking at delaying and peer-led intervention also measured this.

#### Oringanje et al. [52]

All eight papers included in this review measured unintended pregnancy. Three articles looked at skill-building and rates of unintended pregnancy. The remaining five papers individually examined the effect of the following interventions on unintended pregnancy: exposure to parenting, counselling, standard sex education, abstinence and peer-led sessions. Two papers (one peer-led and the other skill-based) also examined childbirth as an outcome of interest.

### Effect of interventions

The results of the interventions to prevent adolescent pregnancy are presented below and in Appendix 3. We report all outcomes reported by the studies within the relevant category. Analyses were then reported as the number of outcomes favouring the intervention out of the total number of outcomes reported, based on the direction of effect and not statistical significance.

#### Skill building

Interventions on skill-building exhibited a propensity for notable efficacy, manifesting improvement across 12 of the 18 distinct outcomes assessed within five studies. Among these interventions, the studies by Lopez et al. [39] and Oringanje et al. [52] stand out, as they summarise the results of one and four studies, respectively. Four cluster-randomised controlled trials (RCTs) and one individual RCT served as the basis for these investigations. Relevantly, the studies mentioned above highlight the positive influence of skill-building interventions on the complex landscape of adolescent pregnancy outcomes. In addition, they cast light on the concurrent improvements observed in attitudes towards contraception and the use of contraceptive methods. Such findings highlight the compelling connection between skill-building interventions and the discernible decline in teen pregnancy rates.

#### Peer-led

Interventions focused on peer-led were generally effective, improving 10 of the 20 outcomes in five studies. Three reviews [39, 43, 52] reported results, and two studies (Kirby [36], Stephenson [65]) were included in two separate reviews. These papers reported that peer-led interventions reduced adolescent pregnancy rates, increased contraceptive use, knowledge, and attitudes towards contraception, and delayed sexual debut rates.

#### Interactive programmes

Interventions focused on interactive programmes generally mixed, improving only two of the four outcomes in one study. One review [39] reported results from one study (cluster RCT). This paper reported that interactive programme interventions improved adolescent pregnancy rates and contraceptive use but did not improve attitudes toward contraception.

#### Delaying sexual debut

Interventions focused on delaying sexual debut were generally ineffective, improving one out of ten outcomes in two review studies. One review [43] reported results from two studies (cluster RCTs). Only one study, Duflo ([22]), measured an improvement in self-reported sexual debut rates.

#### Abstinence

Interventions focused on abstinence were generally effective, improving four out of five outcomes in three studies. Two reviews [43, 52] reported results from two and one study, respectively (three cluster RCTs). One study (Cabezon, [12]) was included in two reviews. These papers reported that interventions that focused on delaying sexual debut improved adolescent pregnancy outcomes and delayed sexual debut but did not improve contraceptive use.

#### Exposure to parental responsibilities

Interventions focused on parenting exposure were generally ineffective, improving one out of three outcomes in one study. One review [52] reported results from one study (individual RCT). This paper found that these interventions improved adolescent pregnancy rates but did not improve contraceptive use.

#### Counselling

Interventions focused on counselling were generally effective, improving two out of two outcomes in one study. One review [52] reported results from one study (individual RCT). This paper found that interventions that provided counselling improved adolescent pregnancy outcomes and improved contraceptive use outcomes.

#### Information only

Interventions focused on only information were mixed, improving one out of two outcomes in one study. One review [52] reported results from one study (individual RCTs). This review indicated that interventions promoted improved adolescent pregnancy rates but did not improve contraceptive use.

### Barriers and enablers in adolescent pregnancy prevention interventions

Table 1 below offers a comprehensive summary of the challenges (barriers) and the supporting factors (enablers) related to interventions specifically designed to address the issue of preventing adolescent pregnancies.

**Table 1:** A summary of some barriers and enablers of interventions focusing on adolescent pregnancy prevention

**Barriers**	**Enablers/facilitators**
• Lack of SRH knowledge, access to adolescent-friendly SRH services and lack of resources to access services.	• Comprehensive and age-appropriate sexual education programmes that provide accurate information about sexual health, contraception and relationships to enable adolescents to make informed decisions.
• Teachers’ lack of sex education training is a barrier to the effective delivery of sex education classes.	• Adolescents can make responsible decisions with the assistance of supportive families, communities and peer networks that promote safe sexual practices and healthy relationships.
• The inability to openly discuss and make contraceptive decisions for adolescent girls, particularly girls with older partners.	• Education and empowerment programmes that empower adolescents through education, life skills training, self-esteem and economic opportunities to reduce the likelihood of adolescent pregnancy by providing alternatives to parenthood.
• Adolescent girls may be subjected to societal pressure to marry and have children once married.	• Implementing interventions that address the economic, socio-cultural and environmental factors that place adolescents in a position to deal with an unintended pregnancy.
	• Encouraging adolescents to delay their sexual debut, countering child marriage practices, increasing uptake and continued use of contraception, and educating girls and boys on the risks of unintended pregnancies.

### The assessment of methodological quality of included reviews

#### Quality of included reviews

The “A MeaSurement Tool to Assess Systematic Reviews 2” (AMSTAR 2) scores for the individual reviews are presented in Appendix 4 [64]. Although the AMSTAR 2 tool is not meant to provide an overall score, we can use it to appraise our confidence in the review results. One review scored high with no or one non-critical weakness [52]. The remaining two reviews scored moderately [39, 43] with one or more non-critical weaknesses. In all three systematic reviews included, namely, [39, 43, 52], it was explicitly stated that the review methods had been established before conducting the review. Moreover, any notable deviations from the established protocol were highlighted and thoroughly explained.

Two reviews used a comprehensive literature search strategy [43, 52]. In the third review, [39] did not search the grey literature or consult with experts in the field. All three reviews listed their excluded studies and justified the exclusion. Only two reviews conducted a meta-analysis [43, 52], both of these reviews adequately justified combining the data in a meta-analysis, and appropriate weighting techniques were used and adjusted for when heterogeneity was detected. All the reviews accounted for the risk of bias in the individual studies when discussing the review results. Two reviews did not investigate publication bias, as there was an insufficient number of trials [39, 43]. Lopez et al. [39] did not conduct a meta-analysis, so there was no investigation into publication bias.

#### Quality of evidence in included reviews

The three reviews used the following to assess the quality of included papers [43, 52] reported using Schünemann’s [63] Grading of Recommendations, Assessment, Development and Evaluations (GRADE) approach [39] applied principles from GRADE and entered the information into a risk of bias table [52] used the GRADE approach to assess the evidence for a reduction in unintended pregnancies to be of moderate quality and low quality for both the contraceptive-promoting interventions (downgraded for imprecision) and for multiple interventions (downgraded for risk of bias, imprecision and inconsistency).

Using the GRADE principles, [39] concluded that the overall quality of evidence is low. Of the five trials included in this review, two were considered very low (Stephenson [65], [69]), another two were considered low (Wight [76], Coyle [18]) and one trial was considered moderate (Kirby [36]) [43] developed a risk of bias summary that assessed nine categories of bias where each paper was graded using a traffic light system. One paper (Cabezon [12]) is at a high risk of bias for six of the nine categories and an unclear risk for the remaining three categories due to inadequate description of methods. Another paper was identified as having a high risk of bias (Cowan [16]). The remaining articles were either designated as low risk of bias or did not provide sufficient information to conclude.

## Discussion

Our search identified 19 studies from three reviews investigating the effectiveness of various sexual health programmes that seek to prevent adolescent pregnancy. The methodological quality of these three reviews was moderate to high, whereas the quality of the individual studies included in the reviews was of low to moderate quality.

### Summary of main results

Although all the interventions included sex education, we grouped the studies using other intervention characteristics, namely delivery strategy (i.e. peer-led, interactive or counselling), key message (abstinence or delaying) or skill-building. Overall, skill-building, counselling and abstinence programmes were generally effective. Interventions that focused on peer-led, information-only and interactive sessions provided mixed results. In contrast, exposure to parenting and delaying sexual debut interventions was generally ineffective. When looking at the groups of outcomes, interventions to prevent adolescent pregnancy were generally effective at adolescent pregnancy outcomes. They provided mixed results for improving contraceptive use, knowledge and delaying sexual debut.

### Overall completeness and applicability of evidence

During this review, we identified studies that fell within each of our intervention groups. However, there were only a few studies within each group. Although all of the studies included described the intervention, these descriptions were not detailed enough to determine the ‘dosage’ and support the comparison of the effect of differences across the studies. Most studies describe the comparison intervention but are not sufficiently detailed to determine their generalisability to other settings.

We faced challenges when categorising the different types of adolescent pregnancy prevention interventions. First, we reviewed the nineteen studies and attempted to identify groups using the review author’s description. All interventions included sex education; therefore, studies were grouped based on additional intervention characteristics. The level of detail in the descriptions varied, and due to unclear descriptions for a handful of interventions, we had to use our judgement to determine group allocation. Consequently, it is not clear how homogenous our groups are. We identified eight different adolescent pregnancy prevention intervention types or groups. All reviews did not contribute data to all categories but did contribute to at least one group.

The studies included in this review were conducted in various global north and south income settings. Thirteen studies were conducted in high-, three in middle-, and three in low-income countries. Of the 19 studies included in the three reviews, four were conducted between 1986 and 1997, ten were between 2002 and 2008, and five were conducted between 2008 and 2015. All of the included reviews are at least 7 years old, and 74% were performed at least 10 years ago. Attitudes to adolescent sexual reproductive health have changed at different rates in different settings. The extent to which changes in attitudes may have influenced the effectiveness of various interventions was not explored in this study but is likely to influence outcomes.

### Quality of evidence

The methodological quality of the three reviews included in this overview was moderate to high. The quality of the individual studies included in the three reviews was of low to moderate quality. Many studies had limited information on intervention fidelity, loss of follow-up or discontinuation. Most of the studies that assessed pregnancy relied upon self-reported rates as one of the assessments. Self-reported pregnancy rates are susceptible to underreporting. Although all included studies presented the effect size for the odds and risk ratios, it was difficult to ascertain the statistical significance for many outcomes as the 95% confidence interval crossed 1.

### Potential biases in the overview process

As this is a rapid review, the search was expedited and may not have identified all potentially important data. If we had more time and resources, we would have increased the number of databases, included the use of grey literature and narrowed the scope. Thus, the review is not without bias. Two reviewers independently assessed all of the studies against the eligibility criteria. We have only included RCTs. Data were extracted from the selected studies, and we evaluated the scientific quality of the individual papers according to AMSTAR 2. The overview only included articles that reported quantitative data on the primary outcome of interest, adolescent pregnancy. Moreover, all of the outcome data are susceptible to self-reporting bias. We did not contact investigators for missing data.

## Authors’ conclusion

Three reviews that used individual trials suggest that adolescent pregnancy prevention interventions that deploy school-based strategies that prevent the occurrence of pregnancies in the first place may effectively reduce unintended adolescent pregnancy rates; improve contraceptive use, attitudes and knowledge; and delay sexual debut. However, the included studies have methodological issues, and our ability to generalise the result is limited. There is a strong desire to reduce unintended adolescent pregnancy rates globally, and schools can provide a valuable platform to reach adolescents and share SRH information. Still, there is little evidence supporting curriculum-based educational programmes alone. These programmes need to be delivered alongside the provision of contraceptives and in a setting where SRH services are readily available and easily accessible. This review found that interventions focused on skill-building and counselling were generally effective at reducing unintended pregnancies. If done well, incorporating peer-led and interactive components into interventions that focus on skill building and counselling can strengthen existing programmes that seek to reduce unintended pregnancies. This review recommends leveraging the school platform and adopting interventions that concurrently deliver skill-building, counselling, contraceptive promotion and sex education. Interventions that emphasise delayed sexual initiation or abstinence must also provide information about contraceptives.

## Appendix 1

**Table:** **Table 2** Search strategy for PubMed

No.	Keywords	Search strategies
1	Adolescent	"Pregnancy in Adolescence"[Mesh] OR Adolescent*[tw] OR teenage*[tw] OR youthful*[tw]
2	Pregnancy	"Pregnancy, Unplanned"[Mesh] OR Unintended*[tw] OR Accidental*[tw] OR Unintentional*[tw] AND "Pregnancy "[MESH] OR Pregnanc*[tw] OR Fertilization*[tw] OR Gestation*[tw] OR Gravidity*[tw] OR ‘pregnan* near (prevent* or interrupt* or terminat*
3	Intervention	Intervention OR strategy OR Solution OR counsel* OR debrief* OR educat* OR teach* OR periodic* OR abstinen* OR sexual* OR abstinen* OR coitus interruptus
4	Combined terms (#1 AND #2 AND #3)	((“Pregnancy in Adolescence” [Mesh] OR Adolescent*[tw] OR teenage*[tw] OR youthful*[tw]) AND (Intervention OR strategy OR Solution OR counsel* OR debrief* OR educat* OR teach* OR periodic* OR abstinen* OR sexual* OR abstinen* OR coitus interruptus)) AND (“Pregnancy, Unplanned” [Mesh] OR Unintended*[tw] OR Accidental*[tw] OR Unintentional*[tw] AND “Pregnancy “[MESH] OR Pregnanc*[tw] OR Fertilisation*[tw] OR Gestation*[tw] OR Gravidity*[tw] OR ‘pregnan* near (prevent* or interrupt* or terminat*))
5	Filter by	“Systematic review”

## Appendix 2

**Table:** **Table 3** List of excluded articles (after a full-text review)

Reviews	Reasons for exclusion
Ampt et al. 2018 [2]	Wrong study population
Aventin et al. 2021 [5]	No numerical data reported on the primary outcome
Biddlecom 2007 [10]	No numerical data reported on the main outcome
Fielding and Williams, 1991 [24]	No numerical data reported on the primary outcome
Kassa et al., 2018 [35]	Does not report data on any of the pre-specified outcomes
Laurenzi et al. 2020 [38]	Interventions do not focus on pregnancy prevention
Morales-Alemán and Scarinci 2016 [47]	No numerical data reported on the primary outcome
Munakamp et al. 2018 [49]	Interventions do not focus on pregnancy prevention
Pradhan et al. 2015 [55]	Interventions do not focus on pregnancy prevention
Rizvi et al. 2020 [57]	Interventions do not focus on pregnancy prevention
Roberts et al. 2021 [58]	Interventions do not focus on pregnancy prevention
Ross et al., 2007 [59]	Not a systematic review
Vanderkruik et al. 2021 [72]	Interventions do not focus on pregnancy prevention
Yakubu 2018 [78]	Interventions do not focus on pregnancy prevention
Arnold 2020 [3]	Insufficient information is provided on the methods
Evans 2020 [23]	Data were not reported on an individual study basis
Gavin et al. 2015 [28]	No numerical data reported on the primary outcome
Maravilla et al. 2017 [42]	No numerical data reported on the primary outcome/wrong study population
McQueston et al. 2013 [45]	No numerical data was reported
Nkhoma et al. 2020 [50]	No numerical data was reported, or the data reported were incomplete in that tests of significance were reported without numerical outcome data
Salam et al. 2016 [60]	Data were not reported on an individual study basis
Taylor et al., 2014 [69]	Not a systematic review
Tolli 2012 [70]	Data were not reported on an individual study basis
Whitaker et al. 2016 [75]	Does not report data on any of the pre-specified outcomes

## Appendix 3

**Table:** **Table 4** Effects of interventions on unintended teen pregnancy outcomes

**Review, year**	**Intervention description and comparisons**	**Outcomes**	**Study (number of participants)**	**Results**	**GRADE or risk of bias assessment results**
**1. Skills building interventions**					
Lopez et al. 2016 [39]	A skills-based HIV, STI and pregnancy prevention curriculum compared to standard school-based activities by community-based agency presenters.	**Use of effective protection against pregnancy at last sex** (condom, oral contraceptive or both)			Low quality
	(Reported by Coyle et al. 2001 [17])	7 months after baseline	(*n*=998)	aOR: 1.62 ± 0.22; *p*=0.03	
		31 months after baseline	(*n*=549)	aOR: 1.76 ± 0.29; *p*=0.05	
		**Condom use at first sex**			Low quality
		7 months after baseline	(*n*=285)	aOR: 0.68 ± 0.48;* p*=0.42	
		31 months after baseline	(*n*=733)	aOR: 1.44 ± 0.27;* p*=0.17	
		**Condom use at last sex**			Low quality
		7 months after baseline	(*n*=1018)	aOR: 1.91 ± 0.27; *p*=0.02	
		31 months after baseline	(*n*=549)	aOR: 1.68 ± 0.25;* p*=0.04	
		**Frequency of sex without a condom in the past 3 months**			Low quality
		7 months after baseline	(*n*=963)	aRM: 0.50 ± 0.31;* p*=0.03	
		31 months after baseline	(*n*=1371)	aRM: 0.63 ± 0.23;* p*=0.05	
		**Attitude towards condoms**			Low quality
		7 months after baseline	*(n*=3510)	aMD: 0.10 ± 0.03; *p*=< 0.01	
		31 months after baseline	(*n*=3751)	aMD: 0.07 ± 0.02; *p*=0.01	
	A school-based intervention combining active learning, information provision and skill development (Reported by Wight et al. 2002 [76])	Unintended pregnancy at 6 months post-intervention	(*n*=2117)	4.0% intervention vs. 3.8% control group	Low quality
		Oral contraception use during last sex at 6 months post-intervention	(*n*=1269)	30.4% intervention vs. 28.0% control group	Low quality
		Condom use at first sex (12 months post-intervention)	(*n*= 2629)	9.7% % intervention vs. 9.1% control group	Low quality
		Condom use at first sex (12 months post-intervention)	(*n*=1269)	44.9% % intervention vs. 44.0% control group	Low quality
Oringanje et al., 2016 [52]	In-person sessions on health/STI education, skills building and contraceptive education	**Unintended pregnancy**	(*n*=484)	*Philliber et al. 2002 *[53] aRR: 0.59 (0.37, 0.94)	Moderate quality
			(*n*=453)	*Howard et al. 1990 *[33] aRR: 0.48 (0.11, 2.09)	Moderate quality
			(*n*=312)	*Kirby et al. 1997 *[36] aRR: 0.86 (0.36, 2.05)	Moderate quality
			(*n*=1887)	*Wight et al. 2002 *[76] aRR: 0.78 (0.51, 1.20)	Moderate quality
**2. Peer-led interventions**					
Lopez et al., 2016 [39]	A school-based peer-led sex education program vs. usual teacher-led sex education (reported by Stephenson et al. [65])	No unintended pregnancy (18 months after intervention)	(*n*=1621)	aOR: 1.40 (0.97 to 2.02)	Very low quality
		Ever had pregnancy (54 months after intervention)	(*n*=1338)	aOR: 0.62 (0.42 to 0.91)	Very low quality
		Ever had unwanted pregnancy (54 months after intervention)	(*n*=1358)	aOR: 0.69 (0.44 to 1.07)	Very low quality
		Ever had an abortion (54 months after intervention)	(*n*=1359)	aOR: 0.56 (0.31 to 1.02)	Very low quality
		Contraception use during first sex (6 months after intervention)	(*n*=230)	aOR: 1.14 (0.81 to 1.62)	Very low quality
		Contraception use during first sex (18 months after intervention)	(*n*605)	aOR: 0.90 (0.73 to 1.11)	Very low quality
		Knowledge of EC pill timing (18 months after intervention)	(*n*=1,784)	aOR: 1.08 (0.78 to 1.50)	Very low quality
		Knowledge of EC pill timing (54 months after intervention)	(*n*=1378)	aOR: 0.93 (0.66 to 1.32)	Very low quality
Lopez et al., 2016 and Oringanje et al., 2016 [39] and [52]	A peer-led intervention focusing on HIV and pregnancy prevention activities (reported by Kirby et al. 1997 [22])	**Oral contraception use at last sex**			Moderate quality
		5 months post-intervention	(n=229)	aOR: 0.73 (0.42, 1.27); p=0.27	
		17 months post-intervention	(*n*=354)	aOR: 0.57 (0.36, 0.91); p=0.02	
		**Condom use at last sex**			Moderate quality
		5 months post-intervention	(*n*=233)	aOR: 0.78 (0.46, 1.34); *p*=0.37	
		17 months post-intervention	(*n*=353)	aOR: 0.76 (0.49, 1.18); *p*=0.22	
		**Pregnancy**			Moderate quality
		5 months post-intervention	(*n*=1402)	aOR: 1.53 (0.66, 3.55); *p*=0.33	
		17 months post-intervention	(*n*=1482)	aOR: 0.82 (0.34, 1.99); *p*=0.66	
		**Knowledge of HIV and pregnancy prevention**			Moderate quality
		5 months post-intervention	(*n*=1460)	MC: 0.59 intervention vs 0.07 control; *p*<0.001	
		17 months post-intervention	(*n*=1529)	MC: 0.89 intervention vs 0.53 control; *p*<0.001	
Mason-Jones et al., 2016 [43]	Peer-led education intervention focuses on HIV prevention, communication, and reproductive health. (Reported by Cowan et al., 2010 [16])	Pregnancy following intervention	(*n*=2586)	aRR: 0.95 (0.72, 1.26)	Moderate quality
		Sexual debut following the intervention	(*n*=2506)	aRR: 1.01 (0.88, 1.15)	Moderate quality
		Condom use at first sex	(*n*=2491)	aRR: 0.98 (0.87, 1.11)	
	Trained peer educators delivered sessions on sexual health topics. (Reported by Stephenson et al. 2008 [65])	Pregnancy	(*n*=4646)	aRR: 0.80 (0.63, 1.03)	Moderate quality
		Condom use at first sex	(*n*=1736)	aRR: 0.98 (0.93, 1.05)	Moderate quality
		Condom use at last sex	(*n*=1550)	aRR: 1.01 (0.93, 1.11)	Moderate quality
**3. Interactive program interventions**					
Lopez et al., 2016 [39]	An interactive program addressing choice, body development, contraception, and parenthood (reported by Taylor et al. [69])	Pregnancy at 4 months after intervention	(*n*=129)	6.3% intervention vs. 4.4% control group	Very low
		Any condom use at 4 months after intervention	(*n*=129)	54.2% intervention vs 36.7% control group; < 0.01	Very low
		Condom use consistency (4-point scale)	(*n*=129)	Mean score: 2.34 ± 1.29	Very low
		Attitude toward teen pregnancy (4-point scale)	(*n*=679)	Mean score: 2.26 ± 0.82	Very low
**4. Abstinence interventions**					
Oringanje et al. 2016 and Mason-Jones et al., 2016 [52] and [43]	In-person sessions on health/STI education, skills building and contraceptive education (reported by Cabezon et al. 2005 [12])	Unintended pregnancy	(*n*=460)	aOR: 0.20 (0.10, 0.39)	Moderate quality
Mason-Jones et al., 2016 [43]	Promoted abstinence until marriage through a teacher-delivered program	Pregnancy	(*n*=365)	aRR: 0.18 (0.08, 0.39)	Low quality
	(Reported by Cabezon et al. 2005 [12])				
	(Reported by Duflo et al. [22])	Pregnancy	(*n*=2754)	aRR: 0.95 (0.72, 1.26)	Moderate quality
		Sexual debut following the intervention	(*n*=6022)	aRR: 0.95 (0.82, 1.09)	Moderate quality
**5. Delaying sexual debut interventions**					
Mason-Jones et al., 2016 [43]	Advised students to delay sexual intercourse and encouraged condom use (Reported by Henderson et al. [30])	Pregnancy	(*n*=4196)	aRR: 1.03 (0.75, 1.40)	Moderate quality
		Sexual intercourse delay	(*n*=2680)	aRR: 0.96 (0.85, 1.10)	Moderate quality
		Condom use at first sex	(*n*=2629)	aRR: 0.99 (0.97, 1.02)	Moderate quality
		Condom use at last sex	(*n*=1269)	aRR: 0.98 (0.88, 1.11)	Moderate quality
	Provided knowledge and skills for delaying sexual debut, reducing risk-taking and improving health service use (Reported by Bonell et al., 2013 [11]	Unintended pregnancy	(*n*=2940)	aRR: 1.06 (0.83, 1.37)	Moderate quality
		Sexual debut delay	(*n=*2940)	aRR: 1.00 (0.93, 1.08)	Moderate quality
		Condom use at last sex	(*n*=2134)	aRR: 1.23 (0.94, 1.61)	Moderate quality
**6. Counselling interventions**					
Oringanje et al., 2016 [52]	In-person sessions on health/STI education, skills building and contraceptive education (Reported by Herceg-Brown et al. 1986 [31])	Unintended pregnancy	(*n*=374)	aRR: 0.96 (0.56, 1.65)	Moderate quality
**7. Exposure to parental responsibilities interventions**					
Oringanje et al., 2016 [52]	In-person sessions on health/STI education, skills building and contraceptive education (Reported by Bonell et al., 2013 [11])	Unintended pregnancy	(*n*=408)	aRR: 0.77 (0.33, 1.79) aRR: 0.50 (0.28, 0.88) aRR: 1.16 (0.88, 1.54)	Moderate quality
**8. Information only interventions**					
Oringanje et al., 2016 [52]	Educational sessions aimed at providing information on sexual risk reduction (reported by Morison-Beedy et al.)	Unintended pregnancy	(*n*=639)		Moderate quality
		Consistent condom use	(*n*=484)		Consistent condom use

## Appendix 4

**Table:** **Table 5** AMSTAR 2 framework review results

	Lopez (2016) [22]	Mason Jones (2016) [25]	Oringanje (2016) [29]
1. Did the research questions and inclusion criteria for the review include the components of PICO?	3	3	4
2. Did the review report explicitly state that the review methods were established before the conduct of the review, and did the report justify any significant deviations from the protocol?	1	1	1
3. Did the review authors explain their selection of the study designs for inclusion in the review?	0	0	1
4. Did the review authors use a comprehensive literature search strategy?	0.5	1	1
5. Did the review authors perform study selection in duplicate?	1	1	1
6. Did the review authors perform data extraction in duplicate?	1	1	1
7. Did the review authors provide a list of excluded studies and justify the exclusions?	1	1	1
8. Did the review authors describe the included studies in adequate detail?	1	1	1
9. Did the review authors use a satisfactory technique for assessing the risk of bias (RoB) in individual studies included in the review?	1	1	1
10. Did the review authors report on the funding sources for the studies included in the review?	0	0	0
11. If meta-analysis was performed, did the review authors use appropriate methods for the statistical combination of results?	N/A	1	0.5
12. If a meta-analysis was performed, did the review authors assess the potential impact of RoB in individual studies on the results of the meta-analysis or other evidence synthesis?	N/A	1	1
13. Did the review authors consider RoB in individual studies when interpreting/discussing the review results?	1	1	1
14. Did the review authors provide a satisfactory explanation for and discussion of any heterogeneity observed in the results?	N/A	1	1
15. If they performed quantitative synthesis, did the review authors conduct an adequate investigation of publication bias (small study bias) and discuss its likely impact on the review results?	0	0	1
16. Did the review authors report any potential sources of conflict of interest, including any funding they received for conducting the review?	1	1	1
Total	11.5	15	17.5

## References

[1] AduBoahen E, Yamauchi C (2017). The effect of female education on adolescent fertility and early marriage: evidence from free compulsory universal basic education in Ghana. J Afr Econ.

[2] Ampt F, Willenberg L, Agius P, Chersich M, Lüchters S, MSC L. Incidence of unintended pregnancy among female sex workers in low-income and middle-income countries: a systematic review and meta-analysis. BMJ OPEN. 2018;8(9). 10.1136/bmjopen-2018-021779. 10.1136/bmjopen-2018-021779 PMC6144321 30224388

[3] Arnold OM, Coyne I. Brief report on a systematic review and meta-analysis of early childhood educational programming and teenage pregnancy prevention. J Adolesc. 2020;84:149–55. 10.1016/j.adolescence.2020.08.008. 10.1016/j.adolescence.2020.08.008 32919364

[4] Aromataris E, Fernandez R, Godfrey CM, Holly C, Khalil H, Tungpunkom P (2015). Summarizing systematic reviews. Int J Evid Based Healthc.

[5] Aventin Á, Gordon S, Laurenzi C, Rabie S, Tomlinson M, Lohan M, et al. Adolescent condom use in Southern Africa: narrative systematic review and conceptual model of multilevel barriers and facilitators. BMC Public Health. 2021;21:1228. 10.1186/s12889-021-11306-6. 10.1186/s12889-021-11306-6 PMC8234649 34172027

[6] de Azevedo WF, Diniz MB, Fonseca ESVB, de Azevedo LMR, Evangelista CB (2015). Complications in adolescent pregnancy: systematic review of the literature. Einstein (Sao Paulo, Brazil)..

[7] Bain LE, Muftugil-Yalcin S, Amoakoh-Coleman M, Zweekhorst MBM, Becquet R, de Cock Buning T (2020). Decision-making preferences and risk factors regarding early adolescent pregnancy in Ghana: stakeholders’ and adolescents’ perspectives from a vignette-based qualitative study. Reprod Health.

[8] Bakilana A. 7 facts about population in Sub-Saharan Africa. Africa Can End Poverty. World Bank Blogs; 2015. 10.4324/9781315879444-17.

[9] Barton K, Redshaw M, Quigley MA, Carson C (2017). Unplanned pregnancy and subsequent psychological distress in partnered women: a cross-sectional study of the role of relationship quality and wider social support. BMC Pregnancy Childbirth.

[10] Biddlecom AE, Munthali A, Singh S, Woog V (2007). Adolescents’ views of and preferences for sexual and reproductive health services in Burkina Faso, Ghana, Malawi and Uganda. Afr J Reprod Health.

[11] Bonell C, Jamal F, Harden A, Wells H, Parry W, Fletcher A, et al. Systematic review of the effects of schools and school environment interventions on health: evidence mapping and synthesis. NIHR Journals Library; 2013. 25642578

[12] Cabezon C, Vigil P, Rojas I, Leiva ME, Riquelme R, Aranda W, et al. Adolescent pregnancy prevention: An abstinence-centred randomized controlled intervention in a Chilean public high school. J Adolesc Health. 2005;36(1):64–9. 10.1016/j.jadohealth.2003.10.011 15661598

[13] Cardona C, Rusatira JC, Cheng X, Silberg C, Salas I, Li Q, Bishai D, Rimon JG (2020). Generating and capitalizing on the demographic dividend potential in sub-Saharan Africa: a conceptual framework from a systematic literature review. Gates Open Res.

[14] Cochrane Effective Practice and Organisation of Care (EPOC). Data collection form. 2022. EPOC Resources for Review Authors. epoc.cochrane.org/resources/epoc-specific-review-authors.

[15] Cowan FM, Pascoe SJ, Langhaug LF, Dirawo J, Chidiya S, Jaffar S, et al. The Regai Dzive Shiri Project: a cluster randomised controlled trial to determine the effectiveness of a multi-component community-based HIV prevention intervention for rural youth in Zimbabwe--study design and baseline results. Trop Med Int Health. 2008;13(10):1235–44. 10.1111/j.1365-3156.2008.02137.x. 10.1111/j.1365-3156.2008.02137.x 18778329

[16] Cowan FM, Pascoe SJ, Langhaug LF, Mavhu W, Chidiya S, Jaffar S, et al. The Regai Dzive Shiri project: results of a randomized trial of an HIV prevention intervention for youth. AIDS. 2010;24(16):2541–52. 10.1097/QAD.0b013e32833e77c9 PMC3058934 20881473

[17] Coyle K, Basen-Engquist K, Kirby D, Parcel G, Banspach S, Collins J, et al. Safer Choices: Reducing teen pregnancy, HIV, and STDs. Public Health Rep. 2001;116(Suppl 1):82–93. 10.1093/phr/116.S1.82 PMC1913682 11889277

[18] Coyle KK, Kirby DB, Robin LE, Banspach SW, Baumler E, Glassman JR. All4You! A randomized trial of an HIV, other STDs, and pregnancy prevention intervention for alternative school students. AIDS Educ Prev. 2006;18(3):187–203. 10.1521/aeap.2006.18.3.187 16774462

[19] Crooks R, Bedwell C, Lavender T (2022). Adolescent experiences of pregnancy in low-and middle-income countries: a meta-synthesis of qualitative studies. BMC Pregnancy Childbirth.

[20] Crowley JL, High AC, Thomas LJ (2018). Desired, expected, and received support: how support gaps impact affect improvement and perceived stigma in the context of unintended pregnancy. Health Commun.

[21] Darroch, J. E., Woog, V., Bankole, A., & Ashford, L. S. Costs and benefits of meeting the contraceptive needs of adolescents. In Adding it up. 2016. https://www.guttmacher.org/sites/default/files/report_pdf/adding-it-up-adolescents-report.pdf.

[22] Duflo E, Dupas P, Kremer M. Education, HIV and early fertility: experimental evidence from Kenya. Am Econ Rev. 2015;105(9):2757–97. 10.1257/aer.20121607 PMC4624413 26523067

[23] Evans R, Widman L, Stokes MN, Javidi H, Hope EC, Brasileiro J. Association of Sexual Health Interventions With Sexual Health Outcomes in Black Adolescents: A Systematic Review and Meta-analysis. JAMA Pediatr. 2020;174(7):676–89. 10.1001/jamapediatrics.2020.0382 PMC7171582 32310261

[24] Fielding JE, Williams CA (1991). Adolescent pregnancy in the United States: a review and recommendations for clinicians and research needs. Am J Prev Med.

[25] Franjić S (2018). Adolescent pregnancy is a serious social problem. J Gynecol Res Obstet.

[26] Garritty C, Gartlehner G, Nussbaumer-Streit B, King VJ, Hamel C, Kamel C, Affengruber L, Stevens A (2021). Cochrane rapid reviews methods group offers evidence-informed guidance to conduct rapid reviews. J Clin Epidemiol.

[27] Gates M, Gates A, Pieper D, Fernandes RM, Tricco AC, Moher D, Brennan SE, Li T, Pollock M, Lunny C, Sepúlveda D, McKenzie JE, Scott SD, Robinson KA, Matthias K, Bougioukas KI, Fusar-Poli P, Whiting P, Moss SJ, Hartling L (2022). Reporting guideline for overviews of reviews of healthcare interventions: development of the PRIOR statement. BMJ (Clinical Research Ed.).

[28] Gavin LE, Williams JR, Rivera MI, Lachance CR. Programs to Strengthen Parent-Adolescent Communication About Reproductive Health: A Systematic Review. AmJ Prev Med. 2015;49(2 Suppl 1):S65–S72. 10.1016/j.amepre.2015.03.022. 10.1016/j.amepre.2015.03.022 PMC10472452 26190849

[29] Hasanpoor E, Hallajzadeh J, Siraneh Y, Hasanzadeh E, Haghgoshayie E (2019). Using the methodology of systematic review of reviews for evidence-based medicine. Ethiop J Health Sci.

[30] Henderson M, Wight D, Raab GM, Abraham C, Parkes A, Scott S, et al. Impact of a theoretically based sex education programme (SHARE) delivered by teachers on NHS registered conceptions and terminations: final result of cluster randomised trial. BMJ. 2007;334(7585):133. 10.1136/bmj.39014.503692.55 PMC1779834 17118950

[31] Herceg‐Brown R, Furstenberg Jr FF, Shea J, Harris KM. Supporting Teenager's Use of Contraceptives: A Comparison of Clinic Services. Fam Plan Perspect. 1986;18(9):61–6. 3792524

[32] Higgins JPT, Thomas J, Chandler J, Cumpston M, Li, T, Page MJ, Welch VA. Cochrane Handbook for Systematic Reviews of Interventions version 6.4 (updated August 2023). 2023. Cochrane. www.training.cochrane.org/handbook. 10.1002/14651858.ED000142 PMC10284251 31643080

[33] Howard M, McCabe JB. Helping Teenagers Postpone Sexual Involvement. Fam Plan Perspect. 1990;22(1):21–7. 2323402

[34] Hubacher D, Mavranezouli I, McGinn E (2008). Unintended pregnancy in sub-Saharan Africa: magnitude of the problem and potential role of contraceptive implants to alleviate it. Contraception.

[35] Kassa GM, Arowojolu AO, Odukogbe AA, Yalew AW (2018). Prevalence and determinants of adolescent pregnancy in Africa: a systematic review and meta-analysis. Reprod Health.

[36] Kirby D, Korpi M, Adivi C, Weissman J. An impact evaluation of Project SNAPP: an AIDS and pregnancy prevention middle school program. AIDS Educ Prev. 1997;9(1 Suppl):44–61. 9083598

[37] Kumar A, Singh T, Basu S, Pandey S, Bhargava V (2007). Outcome of teenage pregnancy. Indian J Pediatr.

[38] Laurenzi CA, Gordon S, Abrahams N, du Toit S, Bradshaw M, Brand A, et al. Psychosocial interventions targeting mental health in pregnant adolescents and adolescent parents: a systematic review. Reprod Health. 2020;17:65. 10.1186/s12978-020-00913-y. 10.1186/s12978-020-00913-y PMC7227359 32410710

[39] Lopez LM, Bernholc A, Chen M, Tolley EE (2016). School-based interventions for improving contraceptive use in adolescents. Cochrane Database Syst Rev.

[40] Malhotra A, Elnakib S (2021). 20 years of the evidence base on what works to prevent child marriage: a systematic review. J Adolesc Health.

[41] Mantell JE, Harrison A, Hoffman S, Smit JA, Stein ZA, Exner TM (2006). The Mpondombili project: preventing HIV/AIDS and unintended pregnancy among rural South African school-going adolescents. Reprod Health Matters.

[42] Maravilla JC, Betts KS, Couto E Cruz C, Alati R. Factors influencing repeated teenage pregnancy: a review and meta-analysis. Am J Obstet Gynecol. 2017;217(5):527–545.e31. 10.1016/j.ajog.2017.04.021. 10.1016/j.ajog.2017.04.021 28433733

[43] Mason-Jones AJ, Sinclair D, Mathews C, Kagee A, Hillman A, Lombard C (2016). School-based interventions for preventing HIV, sexually transmitted infections, and pregnancy in adolescents. Cochrane Database Syst Rev.

[44] Masquelier B, Hug L, Sharrow D, You D, Mathers C, Gerland P, Alkema L, Estimation, U. N. I. G. for C. M (2021). Global, regional, and national mortality trends in youth aged 15–24 years between 1990 and 2019: a systematic analysis. Lancet Glob Health.

[45] McQueston K, Silverman R, Glassman A. The efficacy of interventions to reduce adolescent childbearing in low- and middle-income countries: a systematic review. Stud Fam Plann. 2013;44(4):369–88. 10.1111/j.1728-4465.2013.00365.x. 10.1111/j.1728-4465.2013.00365.x 24323658

[46] Meherali S, Adewale B, Ali S, Kennedy M, Salami BO, Richter S, Okeke-Ihejirika PE, Ali P, da Silva KL, Adjorlolo S, Aziato L, Kwankye SO, Lassi Z (2021). Impact of the COVID-19 pandemic on adolescents’ sexual and reproductive health in low- and middle-income countries. Int J Environ Res Public Health.

[47] Morales-Alemán, M. M., & Scarinci, I. C. Correlates and predictors of sexual health among adolescent Latinas in the United States: A systematic review of the literature, 2004-2015. Prev Med. 2016;87:183–93. 10.1016/j.ypmed.2016.03.005. 10.1016/j.ypmed.2016.03.005 PMC4884463 26972472

[48] Morrison-Beedy D, Jones SH, Xia Y, Tu X, Crean HF, Carey MP. Reducing sexual risk behavior in adolescent girls: results from a randomized controlled trial. The Journal of adolescent health: official publication of the Society for Adolescent Medicine. 2013;52(3):314–21. 10.1016/j.jadohealth.2012.07.005. 10.1016/j.jadohealth.2012.07.005 PMC3580004 23299011

[49] Munakamp L, Ronsmans C, Temmerman M. The social determinants of adolescent pregnancy in sub-Saharan Africa: A systematic review. Soc Sci Med. 2018;213:14–25.

[50] Nkhoma DE, Lin CP, Katengeza HL, Soko CJ, Estinfort W, Wang YC, et al. Girls' Empowerment and Adolescent Pregnancy: A Systematic Review. Int J Environ Res Public Health. 2020;17(5):1664. 10.3390/ijerph17051664. 10.3390/ijerph17051664 PMC7084341 32143390

[51] Noori N, Proctor JL, Efevbera Y, Oron AP (2022). Effect of adolescent pregnancy on child mortality in 46 countries. BMJ Glob Health.

[52] Oringanje C, Meremikwu MM, Eko H, Esu E, Meremikwu A, Ehiri JE. Interventions for preventing unintended pregnancies among adolescents. Cochrane Database Syst Rev. 2016. 10.1002/14651858.cd005215.pub2. 10.1002/14651858.CD005215.pub3 PMC8730506 26839116

[53] Philliber S, Kaye JW, Herlings S, West E. Preventing pregnancy and improving health care access among teenagers: an evaluation of the children's aid society-Carrera program. Perspect Sex Reprod Health. 2002;34(5):244–51. 12392217

[54] Pollock M, Fernandes RM, Pieper D, Tricco AC, Gates M, Gates A, Hartling L (2019). Preferred Reporting Items for Overviews of Reviews (PRIOR): a protocol for development of a reporting guideline for overviews of reviews of healthcare interventions. Syst Rev.

[55] Pradhan R, Wynter K, Fisher J. Factors associated with pregnancy among adolescents in low-income and lower-middle-income countries: A systematic review. J Epidemiol Community Health. 2015;69(9):918–24. 10.1136/jech-2014-205128. 10.1136/jech-2014-205128 26034047

[56] Rasmussen B, Maharaj N, Sheehan P, Friedman HS (2019). Evaluating the Employment Benefits of Education and Targeted Interventions to Reduce Child Marriage. J Adolesc Health.

[57] Rizvi F, Williams J, Bowe S, Hoban E. Factors influencing unmet need for contraception amongst adolescent girls and women in Cambodia. PeerJ. 2020;8:e10065. 10.7717/peerj.10065. 10.7717/peerj.10065 PMC7547592 33083131

[58] Roberts KJ, Smith C, Cluver L, Toska E, Sherr L. Understanding Mental Health in the Context of Adolescent Pregnancy and HIV in Sub-Saharan Africa: A Systematic Review Identifying a Critical Evidence Gap. AIDS Behav. 2021;25(7):2094–107. 10.1007/s10461-020-03138-z. 10.1007/s10461-020-03138-z PMC7810185 33452658

[59] Ross DA, Changalucha J, Obasi AIN, Todd J, Plummer ML, Cleophas-Mazige B, Anemona A, Everett D, Weiss HA, Mabey DC, Grosskurth H, Hayes RJ (2007). Biological and behavioural impact of an adolescent sexual health intervention in Tanzania: a community-randomized trial. AIDS.

[60] Salam RA, Faqqah A, Sajjad N, Lassi ZS, Das JK, Kaufman M, et al. Improving Adolescent Sexual and Reproductive Health: A Systematic Review of Potential Interventions. The Journal of Adolescent Health: official publication of the Society for Adolescent Medicine. 2016;59(4S):S11–S28. 10.1016/j.jadohealth.2016.05.022. 10.1016/j.jadohealth.2016.05.022 PMC5026684 27664592

[61] Santelli J, Rochat R, Hatfield-Timajchy K, Gilbert BC, Curtis K, Cabral R, Hirsch JS, Schieve L (2003). The measurement and meaning of unintended pregnancy. Perspect Sex Reprod Health.

[62] Santhya KG, Jejeebhoy SJ (2015). Sexual and reproductive health and rights of adolescent girls: evidence from low- and middle-income countries. Glob Public Health.

[63] Schünemann H, Brozek J, Guyatt G, Oxman A, editors. GRADE handbook for grading quality of evidence and strength of recommendation; 2011.

[64] Shea BJ, Reeves BC, Wells G, Thuku M, Hamel C, Moran J, Moher D, Tugwell P, Welch V, Kristjansson E, Henry DA (2017). AMSTAR 2: a critical appraisal tool for systematic reviews that include randomised or non-randomised studies of healthcare interventions, or both. BMJ (Clinical Research Ed)..

[65] Stephenson J, Strange V, Allen E, Copas A, Johnson A, Bonell C, et al. The long-term effects of a peer-led sex education programme (RIPPLE): a cluster randomised trial in schools in England. PLoS Med. 2008;5(11):1579–90. 10.1371/journal.pmed.0050224 PMC2586352 19067478

[66] Stoner MCD, Rucinski KB, Edwards JK, Selin A, Hughes JP, Wang J, Agyei Y, Gomez-Olive FX, MacPhail C, Kahn K, Pettifor A. The relationship between school dropout and pregnancy among adolescent girls and young women in South Africa: a HPTN 068 analysis. Health Educ Behav. 2019;46(4):559–68. 10.1177/1090198119831755. 10.1177/1090198119831755 PMC6625926 30819011

[67] Sully EA, Biddlecom A, Daroch J, Riley T, Ashford L, Lince-Deroche N, Firestein L, Murro R. Adding It Up: Investing in Sexual and Reproductive Health 2019. New York: Guttmacher Institute; 2020.

[68] Taylor D, James EA (2011). An evidence-based guideline for unintended pregnancy prevention. J Obstet Gynecol Neonatal Nurs.

[69] Taylor M, Jinabhai C, Dlamini S, Sathiparsad R, Eggers MS, De Vries H (2014). Effects of a teenage pregnancy prevention program in KwaZulu-Natal, South Africa. Health Care Women Int.

[70] Tolli MV. Effectiveness of peer education interventions for HIV prevention, adolescent pregnancy prevention and sexual health promotion for young people: a systematic review of European studies. Health Educ Res. 2012;27(5):904–13. 10.1093/her/cys055. 10.1093/her/cys055 22641791

[71] United Nations, Department of Economic and Social Affairs, & Population Division. World Population Prospects 2019: Highlights (ST/ESA/SER.A/423). In Statistical Papers - United Nations (Ser. A), Population and Vital Statistics Report. UN; 2019. 10.18356/13bf5476-en.

[72] Vanderkruik R, Gonsalves L, Kapustianyk G, Allen T, Say L. Mental health of adolescents associated with sexual and reproductive outcomes: a systematic review. Bull World Health Organ. 2021;99(5):359–373K. 10.2471/BLT.20.254144. 10.2471/BLT.20.254144 PMC8061667 33958824

[73] Wado YD, Afework MF, Hindin MJ (2013). Unintended pregnancies and the use of maternal health services in Southwestern Ethiopia. BMC Int Health Hum Rights.

[74] Wemakor A, Garti H, Azongo T, Garti H, Atosona A (2018). Young maternal age is a risk factor for child undernutrition in Tamale Metropolis, Ghana. BMC Res Notes.

[75] Whitaker R, Hendry M, Aslam R, Booth A, Carter B, Charles JM, et al. Intervention Now to Eliminate Repeat Unintended Pregnancy in Teenagers (INTERUPT): a systematic review of intervention effectiveness and cost-effectiveness and qualitative and realist synthesis of implementation factors and user engagement. Health Technol Assess. 2016;20(16). 10.3310/hta20160 PMC4819203 26931051

[76] Wight D, Raab GM, Henderson M, Abraham C, Buston K, Hart G, et al. Limits of teacher delivered sex education: interim behavioural outcomes from randomised trial. Br Med J. 2002;324(7351):1430. 10.1136/bmj.324.7351.1430 PMC115856 12065268

[77] World Health Organization. Adolescent pregnancy. Fact sheets; World Health Organization; 2023. https://www.who.int/news-room/fact-sheets/detail/adolescent-pregnancy.

[78] Yakubu I, Salisu WJ (2018). Determinants of adolescent pregnancy in sub-Saharan Africa: a systematic review. Reprod Health.

[79] Zulaika G, Bulbarelli M, Nyothach E, van Eijk A, Mason L, Fwaya E, Obor D, Kwaro D, Wang D, Mehta SD, Phillips-Howard PA (2022). Impact of COVID-19 lockdowns on adolescent pregnancy and school dropout among secondary schoolgirls in Kenya. BMJ Glob Health.

